# A Review on Osteoarthritis Knee Management via Contrast Bath Therapy and Physical Therapy

**DOI:** 10.7759/cureus.27381

**Published:** 2022-07-27

**Authors:** Pranali S Fokmare, Pratik Phansopkar

**Affiliations:** 1 Musculoskeletal Physiotherapy, Ravi Nair Physiotherapy College, Datta Meghe Institute of Medical Sciences, Wardha, IND

**Keywords:** weakness, pain, physical therapy, knee osteoarthritis, contrast bath therapy

## Abstract

One of the body's main weight-bearing joints is the knee joint. For this reason, osteoarthritis typically involves it. Osteoarthritis of the knee joint is the condition in which sub-chondral sclerosis of bone occurs, narrowing of joint space is present, and osteophyte formation is seen at the edges of the bone; because of this, there is pain, reduced knee range of motion, and this leads to functional activity limitations. This prevalence is increasing because of a reduced active lifestyle; this is becoming usual in younger populations. Non-pharmacological management of this condition using contrast bath therapy given by different methods like using hot and cold water, towel compression, and smart knee pad device, causes alternate vasodilatation and vasoconstriction helps to reduce pain symptoms. Physical therapy exercises like stretching, strengthening hip and knee muscles, and balance training showed beneficial effects on a range of motion and activities. Articles using keywords were searched on Google Scholar and PubMed, 70 articles were seen of which eight were reviewed that meet the inclusion criteria. Contrast therapy helps to remove metabolic waste by improving blood flow thus reducing pain. Strengthening the hip and knee muscles helps to stabilize the knee joint, and balance exercises help to improve proprioception. From this review, it is concluded that contrast bath therapy is effective in reducing pain when given with different methods of application as compared to individual hot or cold treatment, and given with the help of a device will be more effective than the traditional method. Along with this strengthening of the knee and hip musculature, stretching and balance exercise helps to improve range and functional activity. Exercise and electrotherapy aid in symptom relief and slow the progression rate of this condition.

## Introduction and background

Knee osteoarthritis

To stabilize the body in erect posture important role is played by the knee joint [1]. The knee, which is the biggest synovial joint in humans, is composed of ligaments, a synovial membrane, and osseous structures (the distal femur, proximal tibia, and patella). The avascular cartilage gets lubrication and nutrition from the synovial fluid, which is obtained by the synovial membrane. Unfortunately, this joint suffers a lot of stress and wear, rendering it a frequent location for discomfort conditions such as osteoarthritis (OA) [2]. OA is perhaps the most typical form of arthritis and usually affects the knee. It is primarily caused by aging because it is a progressive disease affecting articular cartilage [3]. Damage to cartilage, a highly specialized connective tissue, is the primary feature of OA. The main causes of OA are aging, genetic susceptibility, metabolic disorders, or trauma. Inflammatory pathways are also activated in cartilage [4]. Knee OA is the most typical chronic condition in older men and women, and it can cause joint pain, lower-limb muscle weakness, and physical dysfunction [5]. Age 45 and above, gender, most commonly females, people overweight or obese, and previous injury to the knee joint are some risk factors. Many people till and above 50 have some changes in the knee joint due to aging, but everyone does not experience related symptoms. Those who show knee OA have decreased working capacity, participation in social gatherings, and reduced life quality [6]. This review aims to see the effect of contrast bath therapy given by different methods and strengthening, stretching, and balance exercises on patients having knee OA.

Pathophysiology of OA

Once thought to be solely a degenerative condition of the cartilage, the latest evidence has shown that OA is a multifactorial disease with a variety of causative factors, including trauma, mechanical forces, inflammation, biochemical reactions, and metabolic disturbances [7]. These structures are impacted as the disease progresses, and changes such as bone remodeling, osteophyte formation, weakness of periarticular muscles, loosening of ligaments, and synovial effusion may become visible [8].

Multiple inflammatory mediators are found in the synovial fluid in OA. Which includes C reactive protein that is considered a marker for the development and progression of the condition, leukotrienes (LKB4), prostaglandins (PGE2), cytokines (TNF, IL1β, IL6, IL15, IL17, IL18, IL21), growth factors (TGFβ, FGFs, VEGF, NGF), nitric oxide and complement factors [9]. All of these components get the ability to locally activate matrix metalloproteinases as well as other hydrolytic enzymes, like cyclooxygenase 2 and prostaglandin E, that could lead to cartilage breakdown due to the destruction of proteoglycan and collagen. In addition to white blood cells, extracellular matrix breakdown causes the release of certain chemicals (damage-associated molecular patterns) that are detected by innate immune cells (macrophages and mast cells), mainly as a protective mechanism. However, such a prolonged and uncontrollable level of inflammation can result in tissue death. The growth of osteophytes, a pathological aspect of OA, has been linked to macrophages in studies on animals [2]. The body also has defense mechanisms at the molecular level, such as growth factors like insulin-like, platelet-derived, fibroblast18, and TGF B, which are unfortunately altered in people with knee OA and may damage the joint [10].

Features of OA

OA patients frequently see their family physician due to pain, which is the disease's predominant symptom. Intermittent pain is typically higher during and after loading exercises. During the whole phase of the disease, inflammatory flares could occur. OA patients also experience stiffness, particularly in the morning, after being sedentary for a while, or in the evening. Unlike the prolonged (often >30 min) stiffness caused by rheumatoid arthritis, this stiffness usually goes away in a matter of minutes. Another reason patients contact their family doctor is due to a loss of mobility and function. Patients describe symptoms that limit them from performing daily tasks like walking, climbing stairs, and doing housework. Depression and sleep issues may be linked to symptoms of OA, which further contribute to disability. OA symptoms reduce a patient's quality of life. The symptoms of OA affect a patient's life quality. OA individuals frequently experience crepitus, a crunching or crackling sound, following passive or vigorous joint movement. Joint deformities are a result of an advanced illness that has damaged the articular capsule, ligaments, muscles, periarticular bone, synovium, cartilage, and periarticular bone [11].

Incidence of OA in knee

The incidence of knee OA was 203 per 10,000 person-years (95% CI, 106-331) in people aged 20 and over, while the global prevalence was 16.0% (95 % CI, 14.3%-17.8%) in people aged 15 and over and 22.9% (95% CI, 19.8%-26.1%) in people aged 40 and over. The ratios of prevalence and incidence in males and females were 1.69 (95% CI, 1.59-1.80, p<0.00) and 1.39 (95% CI,1.24-1.56, p<0.00), respectively. Prevalence and incidence increased with age, peaking at an advanced age on the prevalence and at 70 to 79 years old on incidence. From 2000 to 2020, there was a high prevalence of knee OA globally, which might pose a significant strain on the global health care system. At a continental level Asia (19.2% [95% CI, 15.7%-23.0%]) has high prevalence than in Europe (13.4% [95% CI, 10.1%-17.0%]) and North America (15.8% [95% CI, 11.2%-20.9%]). The major reason that knee OA was more common in Asia as more studies look at radiographic knee OA in Asia than in other countries in the meta-analysis. Asians' habit of bending their knees and squatting, as well as genetic or environmental factors, were other possible reasons [12]. The prevalence of OA in different regions of India varies from 22% to 39%. Indian women aged 65 and above have an approximately 45% prevalence of OA. Until 40, 90% of people show some level of OA; some remain asymptomatic until they get older [13].

Causes of OA

OA is caused according to the etiology. It is of two types: Primary OA is idiopathic or due to non-traumatic conditions and the other type is secondary OA which occurs due to trauma or mechanical misalignment [2]. 

Investigations

For the diagnosis of OA, radiological investigation and symptoms are considered beneficial. The main reason to seek help is the pain. Early changes are seen on x-ray in the form of osteophyte formation and reduced joint space. To find the grade of OA by using x-ray Kellgren and Lawrence scale is used as a tool. According to this scale, grade 1 means there are no bony spurs and space between the joint is normal; in grade 2, there may be a slight narrowing of space and bony spur formation, and in grade 3 narrowing of space between the joint and mild bony spur formation is there and in grade 4 there is moderate bony spur formation a narrowing of space and sclerosis of sub-chondral bone seen [14].

Prevention of OA of knee

To prevent the increasing rate of OA driven by an aging population, rising levels of obesity, and increased physical inactivity, primary and secondary preventative strategies are necessary. Primary strategies include lowering risks, changing behaviors or exposures that increase the risk of developing of disease or increasing resistance to a variety of encounters with a disease agent. Examples of tactics that are pertinent to knee OA are preventing knee injuries and adolescent obesity. Recognizing and treating risk factors for advancement in individuals that are already at risk are examples of secondary prevention. Examples pertinent to knee OA include the identification and monitoring of weight gain and alterations in proprioceptive acuity, dynamic joint stability, and muscle function. People who have already suffered a knee injury can then be managed with weight management and targeted exercise intervention [15].

Physical therapy treatment

One of the non-pharmacological treatments for pain relief is contrast bath therapy. In this therapy, there is the application of heat and cold alternately. This leads to alternate vasodilatation and vasoconstriction of blood vessels, because of which there is an increase in blood flow and removing metabolic waste also helps reduce edema in the affected part, which in turn helps to reduce pain in acute and sub-acute injuries [16]. This therapy helps reduce pain in upper and lower extremities, edema and inflammation of soft tissue, muscle spasm, and subacute phases stiffness in the joints and helps to improve recovery by training. This treatment is easy to use, affordable, non-invasive, and safe [17]. Intra-articular adhesions cause stiffness, as do peri-articular and intramuscular adhesions [18]. Exercise therapy comes in multiple forms and has many systemic and local impacts, which have also been tested in patients with OA. Therapeutic exercise is a broad term that refers to specific physical activities to improve muscular strength, neuromotor control, range of motion, and aerobic fitness. The main aim of the exercise is to improve muscle strength, as commonly weakness is seen in OA patients. Pain is considered a limitation for strengthening dosage. Internal forces of knee pain can be reduced, and physical function can be enhanced with improved lower limb strength. Improved muscle strength may alter biomechanics, leading to a lower joint loading rate or concentrated load in the articular cartilage, delaying the onset and slowing the progression of knee OA. Improved fitness can improve quality of life by expanding the number of daily tasks available, thereby improving physical function [19]. In this review, we are focusing on stretching exercises like hamstring, quadriceps, and calf muscle stretching given in warm up and cool down phase, strengthening exercises for hip and knee flexors and extensors, respectively, and balance exercises given for knee OA patients, and contrast bath therapy’s effect when given with different methods on knee pain in this condition.

Outcome measures are the tools to see the changes pre and post-treatment. To assess pain, visual analog scale is one of the most common tools. It is a 10cm line with no pain at all and the worst pain possible at either end. The participants are asked to mark the level of their pain on it. This distance is measured, which will help you find the difference in pain level before and after the treatment [20]. Thus, goniometry concerns angle measurement primarily angles made by the body's bones at human joints. To assess the restricted ranges of the knee goniometer is used [21]. WOMAC index is one of the reliable tools with three domains: pain, stiffness, and physical function, which is used to find how this OA affects a patient's daily activities [22]. To test the exercise tolerance, instead of a six-minute walk test, a two-minute walk test can be used as it is a reliable measure. It is used to check the walking capacity [23].

## Review

English language articles were searched on Google Scholar and PubMed. Seventy articles were searched from 2018 to date using a combination of search words: knee OA, contrast bath therapy, physical therapy, rehabilitation, pain, and functional disability. Out of 70, eight articles were found relevant in which contrast bath therapy applied with different methods, strengthening, stretching, or balance exercises given to the patients having knee OA were reviewed as below.

A study conducted in 2020 concluded that a contrast bath given by compression method in the elderly age group reduced the pain in the knee joint. They included sixteen elderly people aged between 60 and 74 years. A contrast bath was given with a towel dipped in warm water with a temperature of 41-43˚ C and cold water with 10-18˚ C temperature. Warm compression was applied to the knee joint for 3 min and cold was applied for 1 min, and this alternate warm and cold was given for 20 min every day for seven days. Warm compresses applied to the knee joint will dilate blood vessels, allowing for the restoration of the flow of blood in the compressed area. Heat penetration depth would cause blood vessels to further dilate, which would affect the increase in blood flow. Warm compresses can also induce the hormones endorphins and encephalin to release, which are helpful to reduce pain, improve comfort, and prevent the transfer of pain stimuli. Giving a warm stimulation can reduce tissue damage, according to additional research evidence. Per the findings of earlier studies, applying cold therapy can cause the spleen to be evacuated and can improve circulation and edema reduction through the vasoconstrictive response. Edema reduction can reduce pain by reducing cell necrosis and pressure on pain receptors. Vasoconstriction will result in a decline in the permeability of cellulite, cellular diffusion, and neutrophil migration, which will contribute to reducing inflammation, which impacts pain inhibition [24].

A study to see the effect of cold and contrast hydrotherapy in knee OA patients on outcome was done in 2019. One hundred eighty individuals were selected according to the inclusion criteria. They were randomly divided into two groups. The first group received the cold application for 20 min two times a day, the second group received heating for 1-5 min and cold application for 20 min two times a day, and the telephone number was taken for follow-up at the end of one month. Bio-socio demographic characteristics, 0-10 Numeric pain rating scale, health assessment questionnaire, and World Health Organization Quality of Life (WHOQOL-BRE) were used as outcomes. Considering that OA has a significant negative impact on mental health and quality of life. This implies that individuals with knee OA who experience severe pain and reduced functional abilities too have a low health-related quality of life. So, reduction in pain perception helps to improve health quality. Thus, the group receiving contrast hydrotherapy showed better results than those receiving only cold therapy [25].

A study in 2018 was done to find the effect of hot baths and contrast baths on pain in arthritis patients. Seventy subjects were selected from the age group of 40-60 years and randomly divided into two groups, 35 in each. Group I received a hot bath over the knee and ankle of the patient by pouring water with a temperature of 98-100F for 1 min. And group 2 received a contrast bath using a bucket with hot water at 80-110F, and the affected knee was dipped in it for 45 sec and then in a cold-water bucket with a temperature of 10F for 15-20 sec. And this cycle was repeated three times. The study results showed that the effect of the contrast bath was more significant than the hot bath. In contrast bath therapy alternate vasoconstriction and vasodilatation are leading to an increase in blood flow, along with this, there are lymph vessels contraction and relaxation when exposed to cold and hot, respectively. In comparison to the circulatory system, the lymphatic system lacks a central pump. So this pump helps to remove the stagnant fluid from the affected area and helps in the healing process of the damaged tissue [26].

A study in 2018 was carried out on designing and developing a smart knee pain reduction pad that uses vibration and alternate heating and cooling treatments to relieve pain. Seventy-six subjects were selected according to inclusion criteria aged 45 years. This smart knee pad was connected to the phone, so the patient could efficiently operate it according to their need. Heat and cold combined therapy will be an effective therapeutic option for musculoskeletal illnesses. It will reduce muscle spasms and muscular pain/tension, and increase nerve transmission speed, hence improving range of motion. This technique can also aid athletes with sinusitis, persistent low back discomfort, and muscular sprains [27].

Vibrations help reduce pain by stimulating mechano-receptors and thus block the pain channels at the spinal cord level in the dorsal horn neurons by inhibiting them from sending pain signals to the brain. This helps to reduce pain through this pain gait mechanism [28].

One of the studies in 2019 was done to find the effect of the Otago exercise program in knee OA individuals on balance and fall risk. Time up and go test and berg balance was taken on the first day after the treatment and the fourth week of the treatment. This test shows significant improvement in results pre and post-values. This study concluded that the Otago program increases strengthening and balance and thus reduces the falling risk. Exercises that focus solely on strengthening the lower limbs seem cost-effective in preventing falls. Dynamic and static balance both are essential for walking. The projection of the body's center of mass must be kept within controllable bounds of the base of support, so this ability is crucial. Proper strength and balance training help in improving function and preventing falls [29].

A 2019 study intended to determine the effectiveness of home exercise therapy in increasing the strength of multiple muscles and improving joint flexibility in older adults who had pre-radiographic knee OA using the Kellgren/Lawrence scale. Fifty-two patients completed the trial and were randomly assigned to one of two groups: the multiple exercise group, which included 28 patients, and the control group, which comprised 24 patients. A visual analog scale for knee pain, Japanese Knee OA measures to report physical function, and a hand-held dynamometer to measure the strength of knee extensors are used as outcomes. The study concluded that, rather than simply relying on knee extension muscle power, individuals must adopt home exercise routines that also enhance joint flexibility. In doing daily activities that cause knee pain in people with knee OA, the forces of the hip and knee extensor muscles work in cooperation. As per the study, patients with knee OA had significantly more hip joint extension torque while standing than the control group. To make up for the weak knee extension muscles during knee joint loading, individuals with knee OA are therefore expected to have increased hip muscle activity. Because of this, improvements in knee discomfort and dysfunction occurred in the various exercise group after resistance training of the hip muscles offered stress reduction of the knee extension muscle [30].

A recent study in 2021, was done to find the effect of kinesthesia balance, and agility (KBA) exercises training at different frequencies. One hundred twenty adults were selected and were randomly divided into three groups. Group A received KBA exercises twice a week, group B received KBA exercises thrice a week, and group C received conventional therapy in this patient education for two sessions, then stretching, strengthening, and ultrasound has given for eight weeks. Follow-up was taken at months 3, 4, and 6 post-treatment. This study's findings may provide evidence of the effectiveness of KBA exercise training and the proper number of sessions needed to achieve the highest efficacy in adults with knee OA. There is evidence that the people with knee OA have proprioceptive deficiencies, and that these people benefit from a neuromuscular training program aimed at enhancing proprioception [31].

A study in 2018 was carried out to find the effect of physiotherapy treatment in patients with moderate knee OA having different pain intensities. Sixty participants were selected and randomly divided into three groups according to the pain intensity, that is, mild, moderate, and severe. These groups received pulsed electromagnetic fields, ultrasound, stretching, and strengthening exercises. Visual analog scale, universal goniometer, WOMAC scale, and Jamar hydraulic dynamometer were used as an outcome measure and were evaluated pre and post-intervention at the end of the fourth week. The results showed that the change in pre and post-score was more significant in the moderate pain intensity group than in the other two groups. Stating that pain should be considered while treating knee OA patients. The knee extensors play a significant role in defying gravity, and they experience greater rates of weakening and degeneration than other muscle groups while inactive. Exercise training may be used to reduce the atherogenic suppression of the quadriceps muscles in knee OA patients (Table 1) [32].

**Table 1:** Summary of the studies reviewed on contrast bath therapy and strengthening, balance, and flexibility exercises used for knee osteoarthritis and knee pain patients

Author	Study Type	Study sample	Intervention	Results	Conclusion	Analysis
Rusminingsih et al. 2020 [24]	Experimental	16 elderly people with knee pain	Contrast bath using towel compression	The mean of knee pain before the contrast bath was 5.44, and after it was 3.50. p-value was <0.05	Knee joint pain can be reduced in the elderly using a contrast bath.	The towel compression method may be more effective than the water method.
elFatah et al. 2019 [25]	Quasi-Experimental	180 patients with knee osteoarthritis	Group 1 was given cold therapy using the cold pad. Group 2 was given contrast therapy using a heating pad and cold pad.	Indicators of mean pain decreased. In contrast therapy, it was 3.5 ± 2.1 and in cold therapy, it was 7.0 ± 1.9. The mean HAQ disability index level was higher in group 2 than it was in group1, at 12.7 ± 5.9 and 17.9 ± 6.3, respectively.	When subjects received contrast therapy, there was greater pain reduction and functional improvement.	Hot packs and cold packs can hold more water and can maintain the temperature for a longer period which is the main base of contrast therapy.
Sathyan 2018 [26]	Experimental	70 patients with knee and ankle osteoarthritis	In group 1 hot bath and group 2 got a contrast bath using a bucket.	In study group I, the mean pain perception score was 4.51, and in study group II, it was 3.80. At p 0.05, this calculated t value of6.999* is significant.	The contrast bath had a more significant impact than the hot bath.	Temperature maintenance is a task when giving a contrast bath using water. A constant watch is required in this method to prevent overheating or cooling.
Priya et al. 2018 [27]	Randomized controlled trial	76 individuals with knee pain.	Vibration, alternate heating and cooling, and other smart knee pads are used.	At 0.05 alpha levels, the calculated t exceeds the t critical value. It is highly significant.	Combination therapy using heat and cold will be an effective alternative for treating musculoskeletal disorders, reducing muscle spasms, soreness, and tension in the muscles, as well as speeding up nerve conduction and increasing range of motion.	As the device is used to give contrast bath the main concern of temperature regulation is solved. Along with the additional vibration effect, pain relief is more in this method than the others.
Salekar et al. 2019 [29]	Experimental	Community-dwelling individuals with knee osteoarthritis	Otago exercise program was given	Pre-intervention Berg Balance Scale mean was 36.77, the post-intervention mean was 45.7, P=0.0001, and t=9.466. In the time up and go test, the post-intervention means was28.07, with a P-value of 0.0001 and a t value of17.147. The pre-intervention mean was 40.87.	Otago exercise program is effective in increasing strength and balance.	Otago exercise given in the early stages of OA of the knee will be effective in improving strength and balance.
Suzuki et al. 2019 [30]	Randomized controlled trial	(Multiple exercise group, n =28, control group, n= 24) 52 Community-dwelling elderly individuals with knee osteoarthritis were included.	While the quadriceps muscles were trained in the control group and the knee and hip muscles were trained in the multiple exercise group.	When compared to the control group, JKOM activities of daily living and overall health conditions outcomes significantly improved in the multiple exercise group.	Rather than solely focusing on knee extension muscle strength, it's crucial to design home exercise programs that also aim improving strength and flexibility, and joint flexibility.	As biarticular muscles are present, strengthening of muscles around the hip and knee joint will help to improve strength and balance.
Adhama et al. 2021 [31]	Randomized controlled trial	A total of 120 patients with knee osteoarthritis were included 40 in each group such 3 groups were made.	twice-weekly KBA will be given in group 1, thrice-weekly KBA in group 2, and in the control group conventional physiotherapy given in the ratio of 1:1:1.	Pre-intervention, and after it, and three, four, and six months after the randomization, each group will be assessed.	The results of this study may show how effective KBA exercise training is and how many sessions are needed to have a better effect on people with knee OA.	Kinesthesia, balance, and agility exercises given together will be more effective than given individually in OA patients.
Abdel-Aziem et al. 2018 [32]	Randomized controlled trial	60 subjects with moderate knee osteoarthritis were included. 3 groups were made according to pain intensity that is mild, moderate, and severe.	Exercises involving stretching, strengthening, and pulsed electromagnetic fields were given to 3 groups.	The moderate pain rating group showed a higher change between the pre-and post-score than the other two groups.	One of the significant factors influencing the reduction of knee osteoarthritis pain is the intensity of the pain. As a result, knee osteoarthritis rehabilitation should take pain intensity into account.	In the early stages of OA, pain is the main reason to seek help. So, strengthening the knee extensor from the beginning of symptoms can help to relieve pain and increase the stability of the knee joint.

## Conclusions

Many studies have shown the effect of contrast bath therapy given by traditional method using water or modification of it by using compression method, knee pad device for reducing pain, which is the early complaint of patients because of which there is difficulty in bending the knee and daily activities are hampered. The set temperature ranges of water and the alternate vaso-dilatation and constriction cause the physiological changes in the affected area by increasing the flow of the blood and thus helps to reduce symptoms. The temperature of the water and time of immersion of a part in the water are essential factors in gaining its effect.

The effect of exercise programs on the OA population has also shown positive results. Otago exercise programs, a combination of stretching and strengthening, balance exercises, and electrotherapy, effectively reduce symptoms and thus improve life quality. According to the pain intensity, the effect of these exercise programs varies. Contrast bath therapy is easy to do and, when appropriately explained to the patients, can be applied by them at home bases whenever they experience pain. It is an easy and affordable technique to use in clinical as well as home-based treatment programs.
